# Successful Treatment of Massive Pulmonary Thromboembolism with Reteplase: Case Series

**Published:** 2018-01

**Authors:** Hassan Ghobadi, Zahra Amirajam, Afshin Habibzadeh

**Affiliations:** Department of Internal Medicine, Ardabil University of Medical Sciences, Ardabil, Iran

**Keywords:** Pulmonary Thromboembolism, Reteplase, Bleeding

## Abstract

Massive pulmonary thromboembolism (PTE) has an increased risk of mortality. Thrombolytic therapy is the accepted treatment. Reteplase, a variant of tissue plasminogen activator, has been used in acute myocardial infarction with acceptable safety and efficacy, but studies in massive PTE are rare. In this study we report five cases of successful treatment of massive PTE with reteplase.

## INTRODUCTION

Acute pulmonary thromboembolism (PTE) is a common and potentially lethal form of venous thromboembolism (VTE); PTE typically has a poor prognosis with a high mortality rate despite advances in diagnosis and therapy (1). Acute PTE has been classified into three groups: non-massive or low risk, submassive or moderate/intermediate risk, and massive or high risk. Patients presenting with persistent bradycardia, hypotension, syncope, cardiogenic shock, cardiac arrest, or respiratory failure define as massive PTE (2).

These two groups (massive and submassive PTE) are at increased risk of mortality. The rapid reinstitution of sufficient pulmonary blood flow and right ventricular unloading is important to save the patient’s life (3, 4). Therapeutic methods in patients with massive PTE and hemodynamic collapse include thrombolytic agents, catheter-based thrombus fragmentation or aspiration, and surgical embolectomy (5). Thrombolytic therapy is widely used as a first line treatment with satisfactory results (6).

Recent meta-analysis indicated that thrombolysis was associated with a significant reduction of all-cause mortality. However, following thrombolysis these patients are at risk of major bleeding and fatal or intracranial hemorrhage (7). Different thrombolytic agents have been introduced including alteplase, reteplase and tenecteplase. Reteplase is a variant of tissue plasminogen activator with an extended circulation time, lower fibrin affinity, and greater susceptibility to plasminogen inhibitors (8).

Although the effectiveness of reteplase in acute myocardial infarction is known, few case reports and case studies have reported its utility in acute massive and sub-massive PTE (9–12). Reteplase has been used in most centers to treat massive PTE, but it is not fully approved. In this study, we report a series of 5 cases with massive PTE treated with reteplase.

## MATERIALS AND METHODS

In this retrospective study, five consecutive patients with massive PTE admitted to Imam Khomeini Hospital, Ardabil, Iran, between September 2015 and April 2017 were included. The patients with pulmonary embolism were confirmed by clinical symptoms and CT pulmonary angiography. Massive PTE was defined as sustained hypotension, pulselessness, or persistent profound bradycardia with signs or symptoms of shock in the presence of a newly developed thrombus in the common trunk, right or left main pulmonary artery (2).

All these patients received reteplase 10 units IV bolus over 2 minutes followed 30 minutes later by a second 10 unit IV bolus injection also administered over 2 minutes.

Hospital records of all patients were reviewed for demographic data, predisposing factors, initial clinical presentation, diagnostic studies, hemodynamic status, thrombus location, and outcomes. All patients presented to our emergency department with hemodynamic instability and had tachycardia with S1Q3T3 in electrocardiogram, except case 2 that had normal ECG findings.

### Case 1

The first patient was a 66-year-old female with history of seizure and bedridden for the last three months presenting with hypotension, chest pain and dyspnea. The symptoms begun in the last seven days. Primary echocardiography showed severe RV dysfunction with systolic pulmonary artery pressure (SPAP) of 75 mmHg. The patient received reteplase and during 7 days of admission had no major bleeding or any complications. SPAP had reduced to normal (25 mmHg) in a follow-up echocardiography.

### Case 2

The second case was a 47-year-old male presenting with hypotension, chest pain and dyspnea that had lasted for three days. Doppler sonography showed acute deep vein thrombosis (DVT). Echocardiography showed severe RV dysfunction and RV enlargement with SPAP of 70 mmHg which decreased following reteplase treatment (SPAP=20 mmHg). No complications were noted with reteplase treatment.

### Case 3

The third case was a 64-year-old female who admitted with chest pain and non-ST elevation myocardial infarction; during admission she underwent percutaneous coronary intervention (PCI). Two days following PCI, the patient had persistent chest pain with hypotension and loss of consciousness, and was intubated. Imaging confirmed massive PTE and reteplase was administered. The patient had rectorrhagia in the first 24 hours and cerebellar hemorrhage 36 hours after reteplase administration. Patient was treated conservatively and was extubate in the next three days. Control brain imaging showed no further hemorrhage. Patient was discharged after 10 days with no further complications.

### Case 4

The fourth patient was a 49-year-old male, a known case of COPD and ischemic heart disease with no predisposing factor, presenting with chest pain and vertigo in the last two days. RV enlargement and dysfunction and SPAP of 50 mmHg were reported in the primary echocardiography, which improved in the post treatment follow-up echocardiography with normal RV function. Patient had no complications and discharged after 6 days.

### Case 5

The fifth patient was a 49-year-old male presenting with chest pain during the last two days with highest intensity at the time of visit. The patient had RV enlargement and dysfunction and SPAP of 60 mmHg in echocardiographic study, which improved to 30 mmHg following treatment. Three hours after reteplase administration, the patient had hemoptysis which was managed conservatively. The patient was discharged after 2 days with no complications.

After reteplase treatment, patients received heparin infusion and were discharged with warfarin. Patients were followed for three months and none had any complications during the follow-up period. All patients had follow-up echocardiography a week after discharge that indicated improved PAP and normal RV function. None of the patients died during the hospital stay and follow-up period.

## DISCUSSION

Massive PE is complicated by hemodynamic instability and RV dysfunction which has poor prognosis; therefore, early diagnosis, prompt risk stratification, and aggressive therapeutic strategies are necessary to reduce the mortality rate (1, 2). Rapid restoration of the RV function and pulmonary blood flow will improve patient’s survival (3, 4).

Thrombolytic therapy is the accepted treatment for patients with massive PTE; this therapy significantly improves pulmonary flow and RV function (13). Alteplase is the accepted tPA for the treatment. A few studies have also indicated possible efficacy of reteplase in massive PTE.

We report 5 cases of massive PTE treated successfully with reteplase. All patients had improved echocardiographic parameters, stable hemodynamic and symptoms. Similarly, Liu and Wang (12) reported that reteplase can significantly relieve patients’ symptoms and improve hemodynamic state. Theron and Laidlow (10) also reported improvement in cardiovascular status following reteplase treatment. Tebbe et al. (11) showed significant changes in PAP and hemodynamic parameters following reteplase treatment. Reteplase efficacy in improving PAP and hemodynamic state is also shown in sub-massive PTE (12).

Previous studies have reported that thrombolytic therapy is associated with risk of major bleeding in 13% and of intracranial or fatal hemorrhage in up to 3% of these patients (14–17). Among our patients, one case demonstrated rectorrhagia and cerebellar hemorrhage and one case had hemoptysis. Both patients were managed conservatively and bleeding was controlled.

In the study of Liu and Wang (12), cerebral hemorrhage was seen in one case (5.5%). Zhao et al. (9) also reported one case of thrombolysis-related hemoptysis in their patients with sub-massive PTE. Theron and Laidlow (10) reported no bleeding in their study. Similarly, Tebbe et al. (11) reported no intracranial hemorrhage in their cases.

Previous studies have shown that reteplase has similar risk of bleeding as other fibrin-specific thrombolytic agents (8, 11); Tebbe et al. (11) noted that reteplase has similar safety and efficacy to alteplase in massive PTE.

No mortality was reported among our cases. Similarly, Zhao et al. (9) reported no mortality in hospital and during follow-up. However, 5 cases (27.5%) died in the study of Liu and Wang (12).

## CONCLUSION

In conclusion, reteplase is effective in the treatment of patients with massive PTE and the benefits of reteplase are higher than its risk of bleeding.
